# Fixel‐Based Analysis of Diffusion Imaging as a Quantitative Marker of Disease State in Spinocerebellar Ataxia

**DOI:** 10.1002/acn3.70116

**Published:** 2025-07-15

**Authors:** David J. Arpin, S. H. Subramony, the READISCA Consortium, David E. Vaillancourt, Tetsuo Ashizawa, Alexandra Durr, Thomas Mareci, Thomas Klockgether, Jennifer Faber, Henry L. Paulson, Gülin Öz, Matthew R. Burns

**Affiliations:** ^1^ Department of Applied Physiology & Kinesiology University of Florida Gainesville Florida USA; ^2^ Department of Neurology University of Florida Gainesville Florida USA; ^3^ Norman Fixel Institute for Neurological Diseases, University of Florida Gainesville Florida USA; ^4^ Stanley H. Appel Department of Neurology Weill Cornell Medicine at Houston Methodist Hospital Houston Texas USA; ^5^ Sorbonne Université, Paris Brain Institute – ICM, Inserm, CNRS, APHP, Hopital de la Pitié‐Salpêtrière Paris France; ^6^ German Center for Neurodegenerative Diseases (DZNE) Bonn Germany; ^7^ Department of Neurology University Hospital Bonn Bonn Germany; ^8^ Center for Neurology, Department of Parkinson's Disease, Sleep and Movement Disorders University Hospital Bonn Bonn Germany; ^9^ Department of Neurology University of Michigan Ann Arbor Michigan USA; ^10^ Center for Magnetic Resonance Research, Department of Radiology University of Minnesota Minneapolis Minnesota USA

**Keywords:** early‐stage SCA, neurodegeneration, white matter

## Abstract

**Objective:**

Spinocerebellar ataxias (SCAs) are a group of genetically heterogeneous neurodegenerative diseases causing progressive deterioration and reduced quality of life. Therapeutic advances have been limited by a lack of sensitive anatomic, functional, or diffusion imaging‐based biomarkers. This study aimed to identify white matter differences in the brains of preataxic and early‐stage SCA1 and SCA3 mutation carriers using diffusion magnetic resonance imaging data from a multisite trial setting.

**Methods:**

Fixel‐based analysis was used to estimate microscopic fiber density, macroscopic fiber‐bundle cross‐section, and a combined fiber density and fiber‐bundle cross‐section measure within 45 cerebral and cerebellar tracts. Multivariate ANOVAs compared controls (*n* = 16), pre‐ataxic (*n* = 10 SCA1, *n* = 24 SCA3), and ataxic patients (*n* = 14 SCA1, *n* = 36 SCA3). Clinical variables were correlated with fixel metrics and receiver operating characteristic analyses identified white matter tracts sensitive to distinguishing controls from pre‐ataxic SCA1 and SCA3.

**Results:**

We found widespread white matter deficits in pre‐ataxic and ataxic patients compared to controls with regard to fiber density, fiber‐bundle cross‐section, and combined measures, all of which were associated with clinical measures of ataxia severity. We also found the combined fiber density and fiber‐bundle cross‐section measure from cerebellar tracts distinguished controls from pre‐ataxia with high sensitivity and specificity for both SCA1 (receiver operating characteristic area under the curve = 0.96) and SCA3 (area under the curve = 0.97). The receiver operating characteristic analyses revealed that cerebellar tracts resulted in greater area under the curve than cortico‐spinal and transcallosal tracts.

**Interpretation:**

These results demonstrate that fixel metrics offer sensitive disease‐specific measures of early SCA disease state that correlate with standard clinical measures.

**Trial Registration:**

Clinical Trial Readiness for SCA1 and SCA3 (READISCA), NCT03487367. https://clinicaltrials.gov/ct2/show/NCT03487367.

**Abbreviations**
AUC: area under the curve
CAG: cytosine‐adenine‐guanine
COM: clinical outcome measures
DTI: diffusion tensor imaging
FA: fractional anisotropy
FARS ADL: Friedreich's Ataxia Rating Scale Activities of Daily Living
FBA: fixel‐based analysis
FC: fiber cross‐section
FD: fiber density
FDC: combined measure factoring the effects of fiber density and cross‐section
FDR: false discovery rate
FOD: fiber orientation distribution
ICP: inferior cerebellar peduncle
M1: primary motor cortex
MCP: middle cerebellar peduncle
PMd: dorsal premotor cortex
PMv: ventral premotor cortex
preSMA: pre‐supplemental motor area
ROC: receiver operating characteristic
S1: primary somatosensory cortex
SARA: Scale for the Assessment and Rating of Ataxia
SCA: spinocerebellar ataxia
SCP: superior cerebellar peduncle
SMA: supplemental motor area
SMATT: sensorimotor area tract template
STN to GP: subthalamo‐pallidal tract
TCATT: transcallosal tractography template
TE: echo time
TR: repetition time
WM: white matter

## 1 Introduction

Spinocerebellar ataxias (SCAs) are a group of genetic neurodegenerative diseases resulting in impaired motor control, quality of life, and often early death [1, 2, 3]. There are no effective therapies to slow or stop the progression of the disease, although several candidate therapies are currently in development. One barrier to these potential therapies is a lack of quantitative objective markers of progression across the ataxias [4, 5]. Although traditional anatomic and functional imaging modalities hold promise, results have not been translated into clinical trials or practice [6, 7].

Increasing understanding of pathogenetic mechanisms has led to the threshold of meaningful therapies that hold promise. To test these therapies, accurate measurement of brain neurodegeneration and microstructural damage prior to symptom onset and through disease progression is essential. Over the last two decades, large natural history datasets have characterized clinical outcome measures (COM) but their sensitivity is suboptimal, requiring prohibitively large numbers of patients for clinical trials. Accurate and sensitive biomarkers are urgently needed, and brain imaging holds considerable promise for this purpose.

Diffusion magnetic resonance imaging may provide such a marker, as it allows in vivo assessment of white matter (WM) integrity. WM damage is known to occur in SCA, as histopathological studies show myeline loss in SCA3 patients [8, 9, 10], while animal models of SCA1 [11] and SCA3 [12, 13, 14] show impaired oligodendrocyte maturation with reduced myeline thickness in regions containing fewer mature oligodendrocytes [14]. Recent in vivo studies have utilized diffusion tensor imaging (DTI) to indicate microstructural WM damage in SCA1 and SCA3 [15, 16, 17, 18]. However, DTI measures such as fractional anisotropy (FA) and diffusivity are not fiber‐specific, and their estimation is confounded by the presence of multiple crossing fibers within a voxel. Additionally, DTI cannot separate the intra‐ and extra‐cellular water components within a voxel and cannot be attributed to specific biophysical properties, such as myelin and axonal density [19, 20]. Given that up to 90% of WM voxels may contain crossing fibers [21], higher‐order diffusion models are required to provide additional insights into the properties related to WM degeneration. Fixel‐based analysis (FBA) is one such higher‐order model [22]. FBA estimates microscopic fiber density (FD) by estimating intra‐axonal volume fraction and macroscopic changes in fiber bundle morphology (FC) by estimating fiber‐bundle cross‐section including both intra‐ and extra‐axonal space [22], overcoming the limitations of traditional DTI analysis. Recently, FBA metrics were also shown to have a larger effect size than traditional DTI metrics in a small sample of SCA patients, indicating their potential utility for clinical trials [17].

This study utilized FBA to assess WM damage in presymptomatic and early‐stage SCA1 and SCA3 patients. We applied this analysis to the READISCA dataset, a multinational longitudinal clinical trial readiness study that assessed clinical and imaging features of pre‐ataxic and early‐stage SCA1 and SCA3 mutation carriers (https://clinicaltrials.gov/ct2/show/NCT03487367) [15, 23]. We assess WM damage throughout both the cerebrum and cerebellum, as neuropathological data has revealed widespread damage throughout the brain in SCA [8]. WM integrity was assessed in tracts of interest obtained from WM atlases rather than through a whole‐brain exploratory analysis, providing a replicable approach that can be adopted in future clinical trials. Additionally, we examine the relationship between changes in fixel‐based metrics and clinical measures of ataxia. Finally, we assessed whether WM changes in the cortico‐spinal tracts, transcallosal tracts, cerebellar tracts, or a combination of these can distinguish presymptomatic and early‐stage patients from controls. The results of this analysis may indicate which WM regions could offer the best potential biomarker of disease state for future clinical trials.

## 2 Methods

### 2.1 Participants and Study Design

Participant data was obtained from the National Institute of Mental Health Data Archive (https://nda.nih.gov). Data included 100 subjects from the READISCA clinical trial readiness study (Table 1). Subjects were enrolled at 16 sites across the United States and Europe and included pre‐ataxic, early ataxic SCA1 and SCA3 expansion carriers, and healthy controls [15]. Subjects included in this study consisted of 10 pre‐ataxic SCA1, 14 ataxic SCA1, 24 pre‐ataxic SCA3, 36 ataxic SCA3, and 16 healthy controls (Table 1). SCA gene carriers with a Scale for the Assessment and Rating of Ataxia (SARA) score < 3 were classified as pre‐ataxic, while those with a SARA score ≥ 3 were classified as ataxic [24, 25]. The SARA is an established clinical scale used to quantify ataxia severity [26]. Additional clinical data included cytosine‐adenine‐guanine (CAG) repeat length (long allele) for SCA expansion carriers and Friedreich's Ataxia Rating Scale Activities of Daily Living (FARS ADL) scores. The protocol for the study received prior approval from the appropriate Institutional Review Board, and informed consent was obtained from each subject.

**TABLE 1:** Cohort characteristics and clinical data.

	Control	SCA1 Pre‐ataxic	SCA1 Ataxic	SCA3 Pre‐ataxic	SCA3 Ataxic
*n*	16	10	14	24	36
Sex (female)^c^	7 (43.8%)	7 (70.0%)	9 (64.3%)	17 (70.8%)	18 (50.0%)
Age (years)^c^	43.0 ± 10.1	39.9 ± 7.1	46.2 ± 10.9	37.7 ± 8.2^b^	48.2 ± 9.3^b^
CAG repeat length (long allele)		42.7 ± 1.9	44.6 + 2.4 (*n* = 13)	70.3 ± 2.8	70.3 ± 4.7 (*n* = 35)
SARA	0.63 ± 0.97	0.90 ± 0.88	7.50 ± 2.18	1.00 ± 0.88	6.86 ± 2.34
FARS ADL	0.38 ± 0.62	0.55 ± 0.76^b^	5.36 ± 3.32^a^, ^b^	1.02 ± 2.10^b^	5.01 ± 3.43^a^, ^b^

*Note:* Data represents mean ± SD.

Abbreviations: FARS‐ADL, Friedreich's ataxia rating scale activities of daily living; SARA, scale for the assessment and rating of ataxia.

^a^ *p* < 0.01 for pairwise comparison between Control and Ataxic.

^b^ *p* < 0.01 for pairwise comparison between Pre‐ataxic and Ataxic within a SCA group.

^c^ No significant differences were observed in age and sex between the SCA groups and healthy controls.

### 2.2 Diffusion MRI Acquisition

Diffusion MRI data were acquired using a harmonized protocol on 3T Siemens (Erlangen, Germany) scanners (5 Prisma, 1 Skyra) operating under Syngo MR E11 software and using body coil transmission and a 32‐channel receive array [15]. Prior work has demonstrated that this harmonized protocol can be used to integrate diffusion MRI data acquired across multiple sites and that diffusivity findings were similar with and without the Skyra data and with and without separate correction factors applied to the Skyra data [15]. Multiband acquisition was utilized with opposing phase encoding in the anterior–posterior direction and *q* space sampling split into 2 sets of 98 volumes and 2 sets of 99 volumes, resulting in 2 sets of 197 volumes, including 13 *b* = 0 per phase encode (184 unique diffusion images). Prisma scanners used 1.5 mm^3^ isotropic resolution, repetition time (TR) = 3230 ms, echo time (TE) = 89.2 ms, multiband acceleration = 4, and *b* values = 1500, 3000 s/mm^2^. The Skyra scanner used 1.7 mm^3^ isotropic resolution, TR = 3390 ms, TE = 103.2 ms, multiband acceleration = 4, and b values = 1000, 2000 s/mm^2^. *q* space sampling was the same on Prisma and Skyra scanners, except for b values.

### 2.3 Diffusion MRI Processing

Fixel‐based analysis was performed using MRtrix3 software [22]. Diffusion data were denoised and corrected for susceptibility, motion artifacts, and bias field [27, 28, 29]. Fiber orientation distribution (FOD) was estimated using multi‐shell multi‐tissue constrained spherical deconvolution [30]. Intensity normalization was then performed globally across all subjects [22]. Next, a study‐specific FOD template was created from the group average of all subjects [31, 32, 33], and each individual FOD image was warped to this study‐specific template using a diffeomorphic non‐linear transformation [34]. Apparent FD maps were computed by segmenting each fixel of the FOD images, and log‐transformed fiber cross‐section (FC) maps were calculated from the distortion required when warping the FOD image to the template [22, 35]. A combined measure factoring the effects of fiber density and cross‐section (FDC) was also calculated.

**FIGURE 1:**
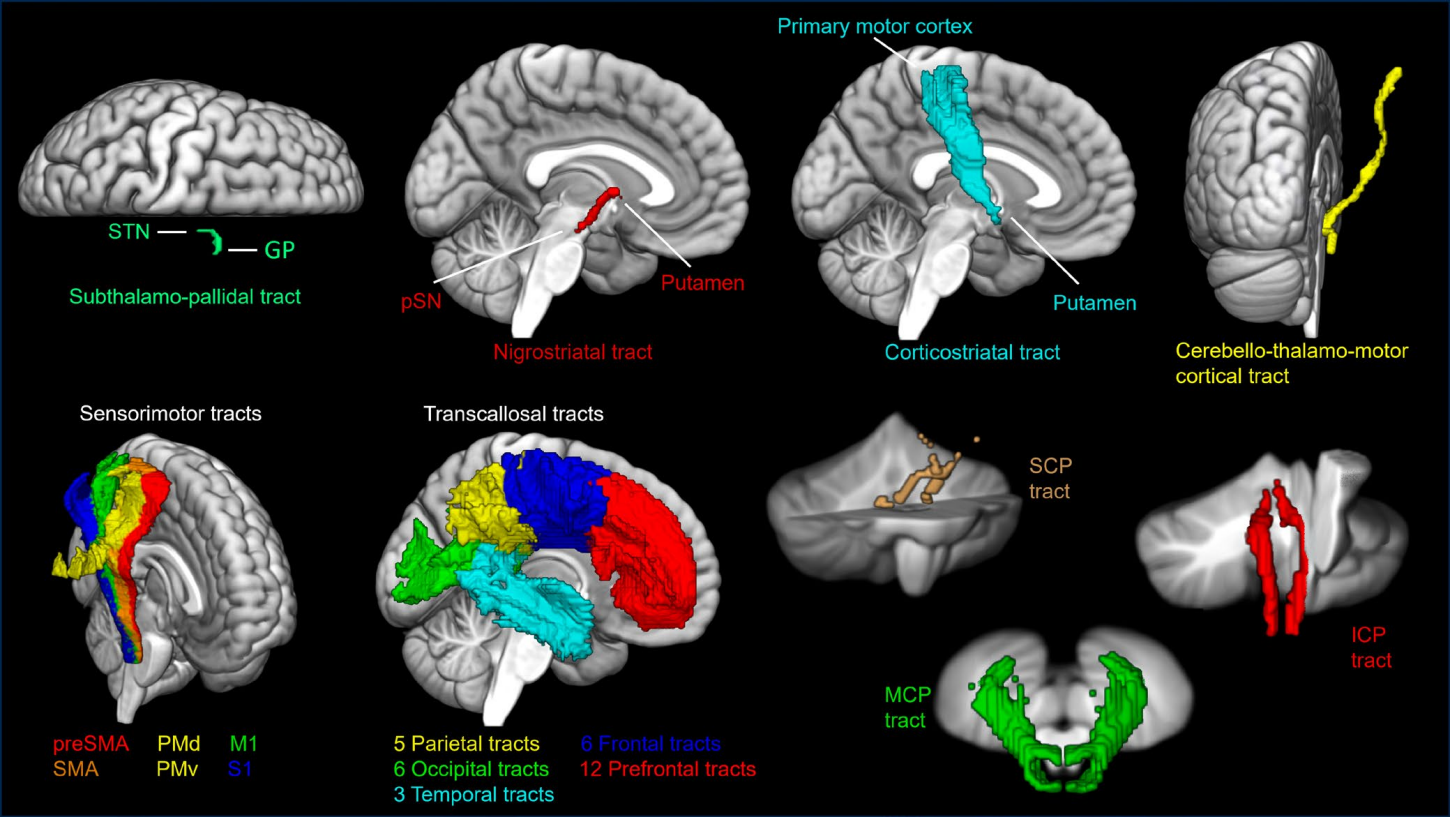
Tracts of interest included a subthalamo‐pallidal tract, nigrostriatal tract, corticostriatal tract, and cerebello‐thalamo‐cortical tract, as well as six sensorimotor tracts from the sensorimotor area tract template, 32 transcallosal tracts from the transcallosal tractography template, and three cerebellar tracts. GP, globus pallidus; ICP, inferior cerebellar peduncle; M1, primary motor cortex; MCP, middle cerebellar peduncle; PMd, dorsal premotor area; PMv, ventral premotor area; preSMA, pre‐supplemental motor area; S1, somatosensory cortex; SCP, superior cerebellar peduncle; SMA, supplemental motor area; STN, subthalamic nucleus.

All three diffusion imaging metrics (FD, FC, FDC) were assessed within 45 WM tracts of interest (Figure 1). Six WM tracts from the sensorimotor area tract template (SMATT) [36], 32 tracts from the transcallosal tractography template (TCATT) [37], 3 tracts from the cerebellar probabilistic white matter atlas [38], and subthalamo‐pallidal (STN to GP), nigrostriatal, corticostriatal, and cerebello‐thalamo‐cortical tracts [39] were used in a tract‐of‐interest analysis. The cortico‐spinal tracts of the SMATT include the primary motor cortex (M1), dorsal premotor cortex (PMd), ventral premotor cortex (PMv), supplemental motor area (SMA), pre‐supplemental motor area (preSMA), and somatosensory cortex (S1) [36]. The TCATT includes 5 parietal tracts, 6 occipital tracts, 3 temporal tracts, 6 frontal tracts, and 12 prefrontal tracts [37]. The superior (SCP), middle (MCP), and inferior (ICP) cerebellar peduncle tracts from the cerebellar atlas were also included [38]. To assess the fixel‐based metrics, the MNI‐space tracts of interest were non‐linearly transformed to the study‐specific population template space by applying a warp obtained from registering the FMRIB FA template to the study‐specific population FA template [27].

### 2.4 Statistical Analysis

Clinical data from control, pre‐ataxic, and ataxic groups were compared using non‐parametric Kruskal‐Wallis or Mann–Whitney U tests. Since SARA scores were used to classify pre‐ataxic and ataxic groups, SARA was compared only between control and pre‐ataxic groups. Group differences (control, pre‐ataxic, ataxic) in fixel‐based measures were assessed using separate multivariate ANOVAs for each measure (FD, FC, FDC), with age as a covariate. Group effects were corrected for multiple comparisons using the False Discovery Rate (FDR) method, and significant variables (P_*FDR*_ < 0.05) underwent FDR‐corrected pairwise tests between the 3 groups (control, pre‐ataxic, ataxic) for the SCA1 and SCA3 cohorts separately. Pearson correlations between clinical variables and those diffusion measures showing significant group effects (P_*FDR*_ < 0.05) were assessed separately for the SCA1 and SCA3 cohorts using only data from the SCA mutation carriers (pre‐ataxic and ataxic).

Separate binary logistic regression analyses were used to distinguish control vs. pre‐ataxic, control vs. ataxic, and pre‐ataxic vs. ataxic groups in the SCA1 and SCA3 cohorts. FDC values for the top three significant tracts (lowest P_*FDR*_ value) from the SMATT, TCATT, and the three cerebellar tracts were entered into separate binary logistic regression analyses for the SCA1 and SCA3 cohorts. To assess whether a combination of cerebellar, SMATT, and TCATT tracts improved classification, binary logistic regression was also performed using the average of the three cerebellar tracts, the average of the three SMATT tracts, and the average of the three TCATT tracts. Data were averaged within each region separately (cerebellar, SMATT, TCATT) to limit the regression to three variables to avoid overfitting the model. Additionally, a binary logistic regression model including the SARA and FARS ALD scores was also assessed for the control vs. pre‐ataxic analysis. This SARA and FARS ADL model was not assessed in control vs. ataxic or pre‐ataxic vs. ataxic regressions since the SARA score was used to define the ataxic groups. Receiver operating characteristic (ROC) analyses were then performed using these regression probabilities and the area under the curve (AUC) was calculated for the SCA1 and SCA3 cohorts. Additionally, the same ROC analysis was performed for the pre‐ataxic vs. ataxic group, combined across both SCA1 and SCA3 cohorts. To determine the 95% confidence interval (CI) for each AUC, a non‐parametric bootstrap with 1000 iterations was performed. At each iteration, the data were resampled with replacement, and the AUC was recalculated. DeLong's test was used to determine significant differences in the AUC between the three tract templates/atlas, the combination of cerebellar, SMATT, and TCATT, and the clinical scores (control vs. pre‐ataxic only) for the SCA1 and SCA3 cohorts.

## 3 Results

Cohort characteristics and clinical data are reported in Table 1. The control group and SCA mutation carriers were age‐ and sex‐matched; however, the ataxic patients were significantly older than pre‐ataxic individuals in the SCA3 cohort (Table 1). The ataxic groups had significantly lower FARS‐ADL scores compared to the control and pre‐ataxic groups for both SCA1 and SCA3 cohorts (Table 1). No significant differences were found between control and pre‐ataxic groups for SARA or FARS‐ADL scores.

Diffusion data indicate widespread WM deficits in pre‐ataxic and ataxic groups compared to controls. The three cerebellar tracts showed significant group effects (P_*FDR*_ < 0.05) across all three diffusion metrics (FD, FC, FDC; ANOVA group effects in Tables 2, 3, 4 respectively). The SMATT, the cerebello‐thalamo‐cortical, and the STN to GP tract showed significant group effects across all three diffusion metrics (P_*FDR*_ < 0.05). Separate follow‐up tests on the SCA1 and SCA3 cohorts revealed these group differences are primarily driven by greater deficits in the ataxic groups. However, Tables 2, 3, 4 show differences exist in all three diffusion metrics between control and pre‐ataxic, as well as pre‐ataxic and ataxic groups for both SCA1 and SCA3 cohorts. The largest differences across all metrics were found in the ICP tract.

**TABLE 2:** Significant FD group and cohort effects.

	ANOVA	SCA1			SCA3		
CON = 16; Pre‐ataxic = 34; Ataxic = 50		Con = 16; Pre = 10; Ataxic = 14			Con = 16; Pre = 24; Ataxic = 36		
	P_*FDR*_	Con vs. Pre	Con vs. Ataxic	Pre vs. Ataxic	Con vs. Pre	Con vs. Ataxic	Pre vs. Ataxic
Cerebellar ICP	**8.94E‐15**	**0.0002**	**3.06E‐09**	**0.011**	**2.72E‐07**	**3.98E‐14**	**0.0006**
Cerebellar MCP	**0.002**	0.135	**0.0002**	0.061	0.565	**0.002**	**0.013**
Cerebellar SCP	**1.11E‐08**	0.123	**5.23E‐07**	**0.0005**	0.192	**1.23E‐06**	**0.0002**
M1‐SMATT	**0.021**	0.235	**0.0002**	**0.009**	0.629	0.058	0.254
PMd‐SMATT	**0.002**	0.103	**0.0001**	**0.026**	0.533	**0.003**	**0.026**
PMv‐SMATT	**0.005**	0.231	**0.0002**	**0.018**	0.683	**0.019**	0.129
SMA‐SMATT	**5.11E‐06**	**0.026**	**1.31E‐05**	**0.018**	0.828	**5.36E‐05**	**0.0003**
preSMA‐SMATT	**0.002**	0.890	**0.002**	**0.001**	0.806	**0.038**	**0.041**
S1‐SMATT	**0.008**	0.323	**0.0001**	**0.006**	0.125	**0.014**	0.553
Cerebello‐thalamo‐cortical	**0.001**	**0.031**	**1.56E‐06**	**0.003**	**0.037**	**0.0007**	0.545
STN to GP	**0.002**	0.172	**0.0007**	0.271	0.232	**0.0008**	0.081
PMd‐TCATT	**0.019**	0.295	**0.0002**	**0.006**	0.647	0.064	0.273
SMA‐TCATT	**0.0008**	0.414	**5.02E‐05**	**0.0009**	0.901	**0.016**	**0.023**
Supramarginal Gyrus‐TCATT	**0.045**	0.441	**0.0006**	0.052	**0.037**	**0.031**	0.857

*Note:* Bold text indicates P_FDR_ < 0.05.

The FD measure shows significant (P_*FDR*_ < 0.05) differences in the three cerebellar tracts between control and ataxic groups for both SCA1 and SCA3 (Table 2). Differences were also found in the ICP tract between control and pre‐ataxic groups for SCA1 and SCA3; however, no differences were found in the MCP or SCP tracts between control and pre‐ataxic SCA1 or SCA3 groups. Apart from the SMA tract in the SCA1 cohort, no differences were found between control and pre‐ataxic groups in the SMATT tracts or the STN to GP tract for SCA1 or SCA3 cohorts. FD measures also show significant (P_*FDR*_ < 0.05) differences in the cerebello‐thalamo‐cortical tract between control and ataxic, and between control and pre‐ataxic in both the SCA1 and SCA3 cohorts. Differences between pre‐ataxic and ataxic groups in the SCA1 cohort, but not the SCA3 cohort, were also found in the cerebello‐thalamo‐cortical tract. This may indicate that the fiber density within this tract is altered primarily during the early stages of disease in SCA3 but throughout disease duration in SCA1.

Results from FC measures show significant (P_*FDR*_ < 0.05) differences in cerebellar ICP and MCP tracts between all three groups for SCA1 and SCA3, while no difference was found in the SCP tract between the control and pre‐ataxic SCA1 group (Table 3). In the SMATT tracts, differences were found between control and ataxic groups for both SCA1 and SCA3 cohorts (P_*FDR*_ < 0.05). Differences were also found between the control and pre‐ataxic SCA3, but not SCA1 groups. No differences were found between pre‐ataxic and ataxic SCA3 groups in the SMATT tracts. Additionally, FC measures from the cerebello‐thalamo‐cortical tract followed this same pattern. This suggests that the fiber‐bundle cross‐section of the sensorimotor tracts may be altered in earlier disease stages for SCA3 and later disease stages for SCA1. Significant differences (P_*FDR*_ < 0.05) were found between all three groups in FC measures in the STN to GP tract for the SCA3 cohort only. This was true for the nigrostriatal tract as well, with the exception of the control and ataxic groups in the SCA1 cohort, which also showed significant differences (P_*FDR*_ = 0.019).

**TABLE 3:** Significant FC group and cohort effects.

	ANOVA	SCA1			SCA3		
CON = 16; Pre‐ataxic = 34; Ataxic = 50		Con = 16; Pre = 10; Ataxic = 14			Con = 16; Pre = 24; Ataxic = 36		
	P_*FDR*_	Con vs. Pre	Con vs. Ataxic	Pre vs. Ataxic	Con vs. Pre	Con vs. Ataxic	Pre vs. Ataxic
Cerebellar ICP	**2.77E‐11**	**0.013**	**4.47E‐06**	**0.013**	**0.0001**	**1.49E‐10**	**0.0003**
Cerebellar MCP	**2.24E‐08**	**0.039**	**2.22E‐05**	**0.023**	**0.005**	**9.29E‐08**	**0.002**
Cerebellar SCP	**1.51E‐08**	0.575	**1.52E‐05**	**0.004**	**0.018**	**1.28E‐07**	**0.0001**
M1‐SMATT	**0.003**	0.211	**0.0009**	0.075	**0.010**	**0.002**	0.240
PMd‐SMATT	**0.002**	0.169	**0.0003**	**0.037**	**0.003**	**0.001**	0.444
PMv‐SMATT	**0.026**	0.513	**0.005**	0.053	**0.050**	**0.022**	0.465
SMA‐SMATT	**0.003**	0.172	**0.0001**	**0.029**	**0.002**	**0.002**	0.840
preSMA‐SMATT	**0.004**	0.698	**0.0002**	**0.008**	**0.005**	**0.002**	0.474
S1‐SMATT	**0.016**	0.513	**0.006**	0.117	0.104	**0.012**	0.204
Cerebello‐thalamo‐cortical	**0.001**	0.194	**0.0005**	0.127	**0.009**	**0.0003**	0.166
STN to GP	**0.0006**	0.632	0.102	0.810	**0.005**	**5.98E‐07**	**0.022**
Nigrostriatal	**0.0002**	0.858	**0.019**	0.264	**0.016**	**3.86E‐06**	**0.014**

*Note:* Bold text indicates P_FDR_ < 0.05.

As expected, the combined FDC measures showed a similar pattern of results as the FD and FC measures, although the FDC measure had the largest number of tracts showing significant (P_*FDR*_ < 0.05) group effects. Similar to FC measures, significant differences were found in cerebellar ICP and MCP tracts between all three groups for SCA1 and SCA3 (P_*FDR*_ < 0.05), while no difference was found in the SCP tract between the control and pre‐ataxic SCA1 group (Table 4). The six SMATT, the cerebello‐thalamo‐cortical, and the TCATT SMA tracts showed significant differences between pre‐ataxic and ataxic groups in the SCA1 cohort (P_*FDR*_ < 0.05), while the SCA3 cohort showed no differences between these groups. This could indicate alterations to these tracts have already occurred in the earliest stages of disease for SCA3. Significant differences (P_*FDR*_ < 0.05) between all three groups were also found for the STN to GP tract, while differences between control and ataxia (P_*FDR*_ = 0.0003), as well as pre‐ataxia and ataxia groups (P_*FDR*_ = 0.039), were found for the nigrostriatal tract in the SCA3 cohort. Means and standard deviations of FD, FC, and FDC metrics are shown in Tables S1–S3 respectively for tracts with significant group effects.

**TABLE 4:** Significant FDC group and cohort effects.

	ANOVA	SCA1			SCA3		
CON = 16; Pre‐ataxic = 34; Ataxic = 50		Con = 16; Pre = 10; Ataxic = 14			Con = 16; Pre = 24; Ataxic = 36		
	P_*FDR*_	Con vs. Pre	Con vs. Ataxic	Pre vs. Ataxic	Con vs. Pre	Con vs. Ataxic	Pre vs. Ataxic
Cerebellar ICP	**2.09E‐16**	**0.0002**	**6.32E‐09**	**0.005**	**4.92E‐07**	**4.74E‐15**	**0.0002**
Cerebellar MCP	**1.33E‐10**	**0.016**	**1.57E‐06**	**0.010**	**0.010**	**7.15E‐10**	**0.0001**
Cerebellar SCP	**1.46E‐10**	0.311	**2.17E‐07**	**0.0005**	**0.029**	**1.24E‐08**	**8.86E‐05**
M1‐SMATT	**0.0002**	0.115	**5.87E‐05**	**0.014**	0.054	**0.0008**	0.126
PMd‐SMATT	**1.81E‐05**	**0.049**	**2.12E‐05**	**0.015**	**0.011**	**6.14E‐05**	0.102
PMv‐SMATT	**0.0001**	0.165	**4.15E‐05**	**0.007**	0.070	**0.0009**	0.113
SMA‐SMATT	**4.93E‐06**	**0.029**	**5.32E‐06**	**0.007**	**0.014**	**2.69E‐05**	0.066
preSMA‐SMATT	**1.81E‐05**	0.502	**1.84E‐05**	**0.0002**	**0.032**	**0.0002**	0.095
S1‐SMATT	**0.0004**	0.195	**0.0001**	**0.019**	0.051	**0.001**	0.187
Cerebello‐thalamo‐cortical	**1.81E‐05**	0.092	**6.42E‐06**	**0.020**	**0.020**	**1.92E‐05**	0.100
STN to GP	**6.04E‐06**	0.204	**0.001**	0.194	**0.031**	**2.11E‐06**	**0.014**
Nigrostriatal	**0.0004**	0.863	**0.012**	0.116	0.200	**0.0003**	**0.039**
Corticostriatal	**0.023**	0.219	**0.0006**	**0.042**	0.072	**0.022**	0.569
SMA‐TCATT	**0.014**	0.232	**0.0002**	**0.013**	**0.019**	**0.016**	0.959
Inferior Temporal Gyrus‐TCATT	**0.042**	**0.037**	**0.005**	0.498	**0.012**	0.251	0.108
Middle Temporal Gyrus‐TCATT	**0.042**	**0.023**	**0.003**	0.488	**0.007**	0.229	0.120

*Note:* Bold text indicates P_FDR_ < 0.05.

SCA mutation carriers (pre‐ataxic and ataxic) revealed significant (P_*FDR*_ < 0.05) negative correlations between CAG repeat length (long allele) and all three diffusion metrics for the SCP tract in SCA1 (Pearson *r* range: −0.67 to −0.76) and SCA3 (Pearson *r* range: −0.49 to −0.55; Tables S4–S6). The SCA1 cohort also showed significant (P_*FDR*_ < 0.05) negative correlations between CAG repeat length (long allele) and all three diffusion metrics for the cerebello‐thalamo‐cortical tract (Pearson *r* range: −0.56 to −0.69) and the FC measure for the ICP tract (*r* = −0.52). Significant (P_*FDR*_ < 0.05) negative correlations were also found between SARA scores and the majority of tracts that showed between‐group effects in each of the diffusion metrics for both SCA1 (Pearson *r* range: −0.42 to −0.81) and SCA3 (Pearson *r* range: −0.31 to −0.68). Significant negative correlations were found between FARS ADL scores and the majority of tracts showing between‐group effects in each diffusion metric for SCA1 (Pearson *r* range: −0.46 to −0.82) and SCA3 (Pearson *r* range: −0.29 to −0.56). In general, correlations with all clinical measures were stronger in SCA1 than in SCA3 cohorts.

**FIGURE 2:**
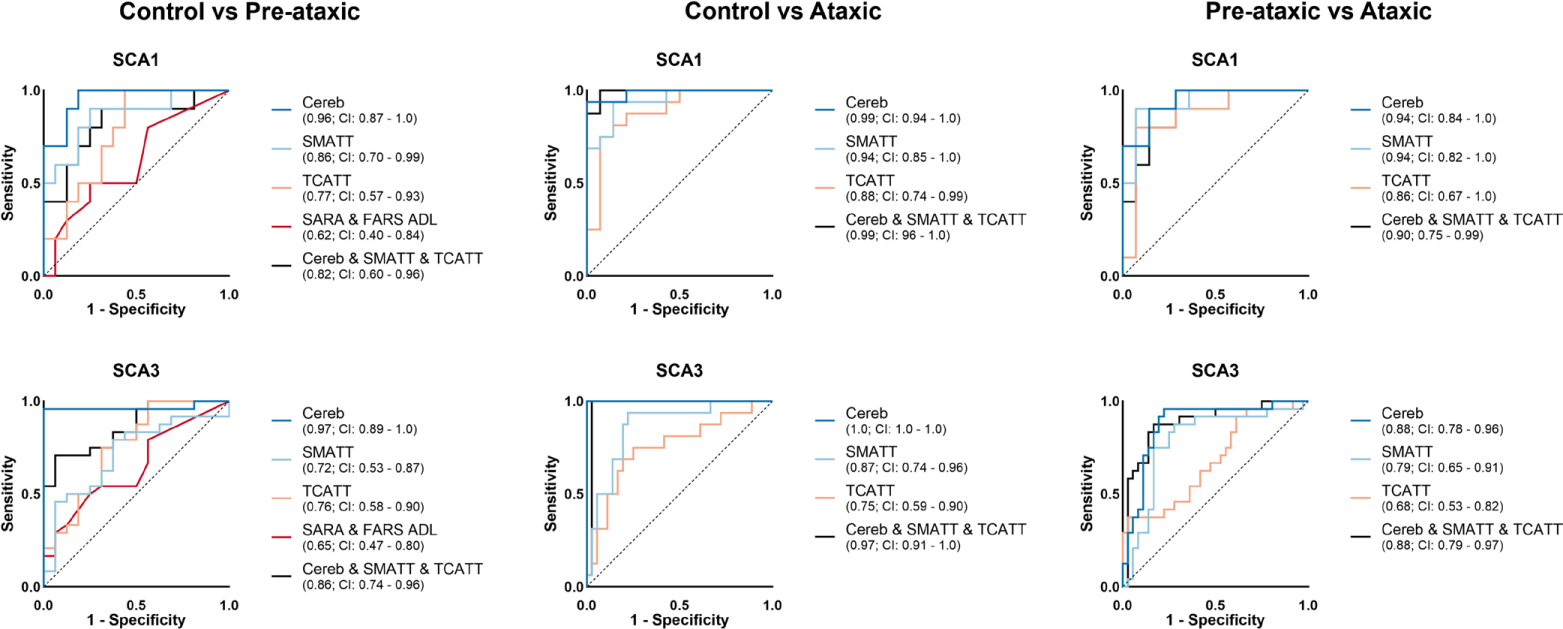
SCA1 (top) and SCA3 (bottom) ROC curves distinguishing control from pre‐ataxic (left), control from ataxic (middle), and pre‐ataxic from ataxic (right). ROC curves were generated using FDC metrics from the 3 cerebellar tracts, the top 3 SMATT tracts, the top 3 TCATT tracts, a combination of the average of the 3 cerebellar, the average of the top 3 SMATT, and the average of the top 3 TCATT tracts, and the SARA and FARS ADL clinical scores. The line of identity is shown as a dotted line (black). The AUC is shown in parentheses within the legend with the 95% confidence interval (CI). Note, the pre‐ataxic vs. ataxic and the control vs. ataxic comparisons do not have ROC curves for the SARA and FARS ADL because the SARA was used to determine the ataxic group.

To assess differences in the ability to distinguish control from pre‐ataxic, control from ataxic, and pre‐ataxic from ataxic groups in the SCA1 and SCA3 cohorts we directly compared the AUCs from the ROC analysis. ROC curves were generated from the top three SMATT tracts (PMd, SMA, preSMA), the top three TCATT tracts (SMA, inferior temporal gyrus, middle temporal gyrus), the three cerebellar tracts (ICP, SCP, MCP), a combination of the average of the cerebellar tracts, the average of the SMATT tracts, and the average of the TCATT tracts. We also compared SARA and FARS ADL clinical scores for the control vs. pre‐ataxic groups only (Figure 2). We used only the FDC diffusion measure for this analysis as it yielded the greatest number of tracts showing between‐group effects, and the FC measure did not show significant between‐group effects in any of the TCATT tracts. The control vs. pre‐ataxic ROC analysis for the SCA1 cohort showed a significantly greater AUC for the cerebellar tracts compared to the TCATT AUC (*p* = 0.040) and clinical scores AUC (*p* = 0.001). In the SCA3 cohort, the control vs. pre‐ataxic ROC analysis showed a significantly greater AUC for the cerebellar tracts compared to the AUCs for the SMATT (*p* = 0.001), TCATT (*p* = 0.011), and clinical scores (*p* = 0.0003), as well as a significantly greater AUC for the combined cerebellar, SMATT, and TCATT tracts compared to the AUCs for the SMATT (*p* = 0.039) and clinical scores (*p* = 0.012). The control vs. ataxic ROC analysis for the SCA3 cohort showed a significantly greater AUC for the cerebellar tracts compared to the SMATT (*p* = 0.013) and TCATT AUCs (*p* = 0.002), as well as a significantly greater AUC for the combined cerebellar, SMATT, and TCATT tracts compared to the SMATT (*p* = 0.039) and TCATT AUCs (*p* = 0.005). The pre‐ataxic vs. ataxic ROC analysis for the SCA3 cohort also showed a significantly greater AUC for the cerebellar tracts compared to the TCATT tracts (*p* = 0.013) and significantly greater AUC for the combined cerebellar, SMATT, and TCATT tracts compared to the TCATT tracts (*p* = 0.003). No significant differences were found between any other AUCs. All AUC comparisons are shown in Table 5 with AUCs, *p*‐values, and *Z*‐values for each comparison.

**TABLE 5:** Comparison of the AUC of ROC curves using DeLong's test.

SCA1				SCA3			
ROC 1 (AUC)	ROC 2 (AUC)	*p*	*Z*‐value	ROC 1 (AUC)	ROC 2 (AUC)	*p*	*Z*‐value
Control vs. Pre‐ataxic							
Cerebellum (0.96)	SMATT (0.86)	0.199	1.28	**Cerebellum (0.97)**	**SMATT (0.72)**	**0.001**	**3.19**
**Cerebellum (0.96)**	**TCATT (0.77)**	**0.040**	**2.05**	**Cerebellum (0.97)**	**TCATT (0.76)**	**0.011**	**2.55**
SMATT (0.86)	TCATT (0.77)	0.447	0.76	SMATT (0.72)	TCATT (0.76)	0.653	−0.45
Cerebellum (0.96)	Combined (0.82)	0.085	1.72	Cerebellum (0.97)	Combined (0.86)	0.069	1.82
SMATT (0.86)	Combined (0.82)	0.649	0.46	**SMATT (0.72)**	**Combined (0.86)**	**0.039**	**−2.06**
TCATT (0.77)	Combined (0.82)	0.668	−0.43	TCATT (0.76)	Combined (0.86)	0.181	−1.34
**Cerebellum (0.96)**	**Clinical (0.62)**	**0.001**	**3.19**	**Cerebellum (0.97)**	**Clinical (0.65)**	**0.0003**	**3.62**
SMATT (0.86)	Clinical (0.62)	0.076	1.77	SMATT (0.72)	Clinical (0.65)	0.559	0.58
TCATT (0.77)	Clinical (0.62)	0.303	1.03	TCATT (0.76)	Clinical (0.65)	0.361	0.91
Combined (0.82)	Clinical (0.62)	0.082	1.74	**Combined (0.86)**	**Clinical (0.65)**	**0.012**	**2.51**
Control vs. Ataxic							
Cerebellum (0.99)	SMATT (0.94)	0.148	1.45	**Cerebellum (1.0)**	**SMATT (0.87)**	**0.013**	**2.49**
Cerebellum (0.99)	TCATT (0.88)	0.121	1.55	**Cerebellum (1.0)**	**TCATT (0.75)**	**0.002**	**3.16**
SMATT (0.94)	TCATT (0.88)	0.243	1.17	SMATT (0.87)	TCATT (0.75)	0.057	1.91
Cerebellum (0.99)	Combined (0.99)	0.730	−0.34	Cerebellum (1.0)	Combined (0.97)	0.318	1.00
SMATT (0.94)	Combined (0.99)	0.155	−1.42	**SMATT (0.87)**	**Combined (0.97)**	**0.039**	**−2.06**
TCATT (0.88)	Combined (0.99)	0.101	−1.64	**TCATT (0.75)**	**Combined (0.97)**	**0.005**	**−2.83**
Pre‐ataxic vs. Ataxic							
Cerebellum (0.94)	SMATT (0.94)	0.897	0.13	Cerebellum (0.88)	SMATT (0.79)	0.071	1.81
Cerebellum (0.94)	TCATT (0.86)	0.402	0.84	**Cerebellum (0.88)**	**TCATT (0.68)**	**0.013**	**2.49**
SMATT (0.94)	TCATT (0.86)	0.457	0.74	SMATT (0.79)	TCATT (0.68)	0.169	1.37
Cerebellum (0.94)	Combined (0.90)	0.309	1.02	Cerebellum (0.88)	Combined (0.88)	0.807	−0.24
SMATT (0.94)	Combined (0.90)	0.481	0.70	SMATT (0.79)	Combined (0.88)	0.067	−1.84
TCATT (0.86)	Combined (0.90)	0.684	−0.41	**TCATT (0.68)**	**Combined (0.88)**	**0.003**	**−2.99**

*Note:* Clinical = SARA score and FARS ALD score. Combined = the average of the 3 cerebellum tracts, the average of the top 3 SMATT tracts, and the average of the top 3 TCATT tracts. Bold text indicates *p* < 0.05.

ROC analysis was also completed for the pre‐ataxic vs. ataxic groups, combined across both SCA1 and SCA3 cohorts. The AUC for the cerebellar tracts was 0.88 (95% CI: 0.80–0.95), the SMATT AUC was 0.81 (95% CI: 0.71–0.90), the TCATT AUC was 0.738 (95% CI: 0.62–0.84), and the AUC for the combined cerebellar, SMATT, and TCATT tracts was 0.88 (95% CI: 0.80–0.95). A significantly greater AUC was found for the cerebellar tracts compared to the TCATT AUC (*p* = 0.022), as well as a significantly greater AUC for the combined cerebellar, SMATT, and TCATT tracts compared to the TCATT AUC (*p* = 0.010).

## 4 Discussion

This study demonstrates that FDC measured in the three cerebellar tracts distinguishes controls from pre‐ataxic, controls from ataxic, and pre‐ataxic from ataxic patients with high sensitivity and specificity for both SCA1 and SCA3. The cerebellar tracts performed better than or equal to all other tracts when distinguishing between groups and across cohorts. The ICP tract also shows the largest difference across all metrics and is the only tract that significantly differed between all three groups for both SCA1 and SCA3 cohorts across the three fixel‐based metrics. Additionally, all three fixel‐based metrics are associated with clinical measures of ataxia. These findings suggest that fixel‐based measures from the cerebellar tracts may offer significant utility as a biomarker for future clinical trials.

These findings extend prior work by demonstrating that the cerebellar tracts together (ICP, MCP, SCP) distinguish controls from pre‐ataxic patients with slightly higher sensitivity and specificity for SCA1 (AUC = 0.96) and SCA3 patients (AUC = 0.97) compared to previous studies that demonstrated the right ICP distinguished control and pre‐ataxic patients using FA for SCA1 (AUC = 0.91) and radial diffusivity for SCA3 (AUC = 0.92) [15]. Furthermore, the current study directly compares measures of WM damage in the cerebrum and cerebellum of pre‐ataxic and early‐stage SCA1 and SCA3 patients. We found widespread WM deficits in both pre‐ataxia and ataxia patients in cortical, basal ganglia, and subcortical to cortical tracts. All six tracts of the SMATT, the cerebello‐thalamo‐cortical tract, and the STN to GP tract show significant group effects across all three fixel‐based metrics. Although largely driven by differences between the control and ataxic groups, there are pairwise differences across all cohorts in both diseases. Importantly, the cerebellar tracts significantly outperformed the TCATT tracts and clinical scores for distinguishing control and pre‐ataxic SCA1 patients when directly comparing ROC curves. In SCA3, the cerebellar tracts significantly outperformed the SMATT tracts, TCATT tracts, and clinical scores when distinguishing control and pre‐ataxic patients. Although ROC curves for the cerebellar tracts were not significantly better for every comparison, they did show greater AUCs relative to all other tracts for all comparisons except the control vs. ataxic SCA1 cohort and pre‐ataxic vs. ataxic SCA3 cohort, which resulted in equal AUCs between the cerebellar tracts and the combined cerebellar, SMATT, and TCATT tracts. Despite the relative performance of the ROCs, this broader analysis remains potentially valuable in a greater set of clinical research contexts. SCAs are diverse and phenotypically complex diseases resulting in degeneration of multiple cortical and subcortical tracts. Currently available clinical metrics are relatively limited and crude measures of disease state and progression, and as a result, there is significant value in a non‐invasive biomarker that is both highly sensitive and able to broadly sample functionally relevant WM tracts. An example of this potential utility is represented in the difference between the STN to GP tract seen in SCA3, which is known to display clinical symptoms of parkinsonism to a greater extent than SCA1.

One notable strength of this study is that FBA provides information about the underlying pathophysiologic changes in WM. Our results demonstrate micro‐ and macro‐structural WM deficits are present before symptom onset for both SCA1 and SCA3 mutation carriers. Recent work by Chandrasekaran et al. has also utilized the READISCA dataset to demonstrate that DTI metrics are altered at the pre‐ataxic stage in SCA1 and SCA3, suggesting WM damage [15]. However, altered DTI measures can indicate various pathological abnormalities such as myelin or axon degeneration, altered fiber organization, or a combination of biological processes [19, 20, 21]. The current study provides additional insight into the processes contributing to WM damage in SCA by assessing microscopic fiber density and macroscopic changes in fiber bundle morphology [22]. Our results suggest that across all fixel‐based metrics, widespread WM damage was already present in the pre‐ataxic SCA3 cohort, while fewer tracts were altered in pre‐ataxic SCA1. This is consistent with prior work suggesting SCA1 is characterized mainly by atrophy in the pons and cerebellum [40], while SCA3 shows atrophy throughout the cerebrum and cerebellum [41]. Additionally, in the pre‐ataxic SCA3 cohort, reduced FC is found in the majority of WM tracts while FD remained relatively preserved, particularly in the MCP and SCP, SMATT, cerebello‐thalamo‐cortical, and STN to GP tracts. These findings may indicate axonal atrophy or demyelination, which could potentially result in decreased fiber cross‐section while axonal density remains unchanged. Myelin loss in SCA3 patients has previously been demonstrated in several histopathological studies [8, 9, 10]. Impaired oligodendrocyte maturation has also been shown in mouse models of SCA1 [11] and SCA3 [12, 13, 14], with reduced myelin thickness found in regions containing fewer mature oligodendrocytes [14]. Moreover, dysfunction in oligodendrocyte maturation occurs early in SCA3 pathogenesis [13] further supporting the hypothesis that reduced FC found in the pre‐ataxic SCA3 cohort may represent a reduction in myelin thickness prior to axonal degeneration. Finally, we also found that the ICP tract shows the greatest reduction in pre‐ataxic patients for both SCA1 and SCA3 and is the only tract that is significantly different for all fixel‐based metrics. This may suggest that both demyelination and axonal degeneration have already occurred within the ICP tract before the onset of ataxia symptoms, indicating that this tract may show the earliest signs of disease and could provide a robust metric for future clinical trials. This hypothesis is further supported by traditional DTI metrics that previously identified the ICP as an important region for distinguishing controls from pre‐ataxic individuals, suggesting alterations in spinal input to the cerebellum occur early in the disease process [15]. In addition to alteration in the cerebellar peduncles, spinal cord atrophy in pre‐ataxic individuals led to the proposal of a caudal‐rostral progression of SCA3 pathology [16]. A caudal‐rostral progression may also explain why the cerebellar tracts perform best when distinguishing control from pre‐ataxic individuals in the current study.

When comparing pre‐ataxic and ataxic patients, our results show reduced FD, FC, and FDC in all cerebellar tracts for ataxic SCA1 and SCA3 (apart from the MCP tract in SCA1 which showed a trend but not a significant difference [P_*FDR*_ = 0.061]). This suggests WM damage within the cerebellar tracts may progress as patients go from pre‐ to early‐stage ataxia, although longitudinal data is needed to confirm this. In the SCA1 cohort, FD is reduced in cerebello‐thalamo‐cortical and all SMATT tracts in ataxia compared to pre‐ataxia, while FC is reduced only in PMd, SMA, and preSMA SMATT tracts. These findings could indicate greater microstructural alterations, such as axonal degeneration, may be occurring in early‐stage ataxic SCA1 patients while the overall fiber cross‐section is less affected, potentially due to axonal swelling known to occur in SCA [8]. In the SCA3 cohort, PMd, SMA, and preSMA SMATT tracts show reduced FD in ataxia compared to pre‐ataxia, while no differences in FC are found in these tracts, likely because the reduction in FC had already taken place in the pre‐ataxia stage. As stated above, this may suggest demyelination during the pre‐ataxia stage in SCA3, with axonal degeneration beginning to occur in early‐stage ataxia.

In early‐stage symptomatic ataxia patients, we found reduced FD, FC, and FDC compared to controls in all three cerebellar tracts for both SCA1 and SCA3 cohorts. Prior DTI studies have also reported altered FA and diffusivity measures within the cerebellar peduncles, supporting our findings [15, 16, 18]. We also found a widespread reduction in all fixel‐based metrics within a number of tracts throughout the cerebrum, particularly through cerebello‐thalamo‐cortical and SMATT tracts, for both SCA1 and SCA3 cohorts. Adanyeguh et al. previously showed decreased FD, FC, and FDC in the corticospinal tract of SCA1 and SCA3 patients compared to controls [17]. Additionally, damage to cerebral WM tracts has been reported in more advanced stages of SCA1 and SCA3 [16, 42]. Overall, our results suggest widespread micro‐ and macrostructural WM damage has already occurred in early‐stage SCA1 and SCA3 patients, despite prior reports that atrophy was typically limited to the cerebellum in SCA1 [40].

The correlations between all SCP fixel‐based metrics and CAG repeat length might suggest greater WM damage in SCA1 and SCA3 patients with longer CAG repeat lengths. The correlations between both SARA and FARS ADL scores and the majority of tracts that showed between‐group effects in each of the diffusion metrics may also suggest greater WM damage for SCA1 and SCA3 patients with worse clinical symptoms. FDC measures have previously shown negative correlations with SARA scores, although these patients had slightly higher SARA scores and did not include pre‐ataxic patients [17]. In general, correlations with all clinical measures were stronger in SCA1 than in SCA3 cohorts, potentially due to the faster progression often seen in SCA1 [40], or the phenotypic heterogeneity of SCA3 [43]. Importantly, these correlations demonstrate fixel‐based measures of WM damage are associated with COMs despite the limited range in clinical scores due to the focus on presymptomatic and early‐stage ataxia.

Overall, these results demonstrate that the cerebellar tracts perform well when distinguishing between control and SCA patients, even in the pre‐ataxic stage, and may provide a strong metric for testing disease‐modifying treatments. These FBA metrics may also be sensitive to progression effects, with WM tracts outside the cerebellum potentially becoming more involved as the disease progresses, although further analysis of longitudinal data is required to confirm this possibility. As disease‐modifying therapies for ataxias move through clinical trials, effective biomarkers for progression in early and pre‐symptomatic disease become critical to efficient and sensitive assessment of efficacy among therapeutic agents. Data presented here support the use of FBA metrics for sensitive and potentially disease‐specific measures of early disease state that correlate with standard clinical measures. Future longitudinal studies are needed to determine the ability of FBA metrics to track disease progression, however, these initial findings demonstrated the potential of these metrics as a noninvasive marker of WM integrity that can enhance future clinical trials.

## Author Contributions

S.H.S., D.E.V., and M.R.B. contributed to the design of the study. D.J.A. contributed to the analysis of the data and preparing the figures. D.J.A. and M.R.B. contributed to drafting the manuscript. All authors reviewed the manuscript. T.A., G.Ö., H.L.P., A.D., J.F., T.M., and T.K. contributed to the conception and design of the READISCA study and the acquisition of clinical and imaging data. Members of the READISCA Consortium contributed to the collection of data (https://readisca.org/readisca‐team/).

## Conflicts of Interest

T.K. has received consulting fees from Biogen, UCB, and Vico Therapeutics, which are developing therapeutics for SCAs. T.A. has received grants from Biogen and participates in Biohaven clinical trials. G.Ö. consulted for UCB Biopharma SRL/Lacerta Therapeutics Inc. and Institut de Recherches Servier and receives research support from Biogen, which develops therapeutics for SCAs. J.F. has received consulting fees from Vico Therapeutics, which develops therapeutics for SCAs.

## Supporting information


Data S1.

## Data Availability

Data used in the preparation of this manuscript were obtained from the National Institute of Mental Health (NIMH) Data Archive (NDA). NDA is a collaborative informatics system created by the National Institutes of Health to provide a national resource to support and accelerate research in mental health. Dataset identifier(s): (DOI: 10.15154/8hwd‐8q58). This manuscript reflects the views of the authors and may not reflect the opinions or views of the NIH or of the Submitters submitting original data to NDA.
