# Supplementary material for: Fixel‐Based Analysis of Diffusion Imaging as a Quantitative Marker of Disease State in Spinocerebellar Ataxia
Source: Ann Clin Transl Neurol. 2025 Jul 15;12(9):1846–57. doi: 10.1002/acn3.70116 (PMC12455865; doi:10.1002/acn3.70116)
Supplement: Supplementary file 1 — Data S1. [file ACN3-12-1846-s001.docx]

**Supplemental Material**

Supplemental Table 1: FD mean (SD) of white matter tracts showing significant group effects.

|  | Con  (n = 16) | Pre  (n = 34) | Ataxic  (n = 50) | SCA1 Pre  (n = 10) | SCA1 Ataxic  (n = 14) | SCA3 Pre  (n = 24) | SCA3 Ataxic  (n = 36) |
| --- | --- | --- | --- | --- | --- | --- | --- |
| Cerebellar ICP | 0.543 (0.03) | 0.485 (0.03) | 0.447 (0.03) | 0.493 (0.03) | 0.451 (0.03) | 0.482 (0.03) | 0.446 (0.03) |
| Cerebellar MCP | 0.535 (0.02) | 0.531 (0.03) | 0.505 (0.03) | 0.523 (0.03) | 0.494 (0.03) | 0.534 (0.03) | 0.509 (0.03) |
| Cerebellar SCP | 0.708 (0.04) | 0.683 (0.03) | 0.638 (0.05) | 0.680 (0.03) | 0.606 (0.05) | 0.684 (0.03) | 0.651 (0.05) |
| M1-SMATT | 0.565 (0.03) | 0.561 (0.02) | 0.540 (0.03) | 0.554 (0.02) | 0.523 (0.02) | 0.564 (0.03) | 0.546 (0.03) |
| PMd-SMATT | 0.521 (0.02) | 0.516 (0.02) | 0.493 (0.03) | 0.507 (0.02) | 0.478 (0.03) | 0.519 (0.03) | 0.499 (0.02) |
| PMv-SMATT | 0.498 (0.02) | 0.493 (0.02) | 0.474 (0.02) | 0.487 (0.02) | 0.457 (0.03) | 0.496 (0.02) | 0.480 (0.02) |
| SMA-SMATT | 0.565 (0.02) | 0.559 (0.02) | 0.526 (0.03) | 0.546 (0.02) | 0.512 (0.03) | 0.565 (0.03) | 0.532 (0.02) |
| preSMA-SMATT | 0.524 (0.02) | 0.527 (0.02) | 0.504 (0.02) | 0.525 (0.02) | 0.494 (0.02) | 0.528 (0.02) | 0.508 (0.02) |
| S1-SMATT | 0.541 (0.02) | 0.531 (0.02) | 0.513 (0.03) | 0.533 (0.02) | 0.497 (0.03) | 0.531 (0.03) | 0.518 (0.03) |
| Cerebello-thalamo-cortical | 0.578 (0.02) | 0.556 (0.03) | 0.543 (0.03) | 0.556 (0.01) | 0.524 (0.03) | 0.555 (0.03) | 0.550 (0.03) |
| STN to GP | 0.569 (0.06) | 0.540 (0.07) | 0.488 (0.07) | 0.526 (0.08) | 0.485 (0.06) | 0.546 (0.06) | 0.490 (0.07) |
| PMd-TCATT | 0.525 (0.02) | 0.522 (0.02) | 0.503 (0.03) | 0.516 (0.02) | 0.489 (0.02) | 0.524 (0.02) | 0.508 (0.02) |
| SMA-TCATT | 0.579 (0.03) | 0.581 (0.03) | 0.549 (0.03) | 0.571 (0.02) | 0.532 (0.02) | 0.585 (0.03) | 0.556 (0.02) |
| Supramarginal Gyrus-TCATT | 0.514 (0.02) | 0.502 (0.02) | 0.494 (0.02) | 0.506 (0.03) | 0.485 (0.01) | 0.501 (0.02) | 0.497 (0.02) |

Supplemental Table 2: FC mean (SD) of white matter tracts showing significant group effects.

|  | Con  (n = 16) | Pre  (n = 34) | Ataxic  (n = 50) | SCA1 Pre  (n = 10) | SCA1 Ataxic  (n = 14) | SCA3 Pre  (n = 24) | SCA3 Ataxic  (n = 36) |
| --- | --- | --- | --- | --- | --- | --- | --- |
| Cerebellar ICP | 0.115 (0.09) | 0.006 (0.08) | -0.131 (0.11) | 0.027 (0.07) | -0.128 (0.13) | -0.004 (0.08) | -0.131 (0.10) |
| Cerebellar MCP | 0.160 (0.10) | 0.069 (0.10) | -0.095 (0.14) | 0.082 (0.09) | -0.102 (0.17) | 0.064 (0.11) | -0.093 (0.13) |
| Cerebellar SCP | 0.059 (0.08) | 0.005 (0.09) | -0.128 (0.11) | 0.037 (0.10) | -0.144 (0.12) | -0.009 (0.08) | -0.122 (0.11) |
| M1-SMATT | 0.056 (0.08) | 0.012 (0.06) | -0.045 (0.09) | 0.021 (0.07) | -0.069 (0.10) | 0.008 (0.05) | -0.036 (0.09) |
| PMd-SMATT | 0.076 (0.08) | 0.019 (0.07) | -0.040 (0.10) | 0.036 (0.06) | -0.060 (0.09) | 0.012 (0.07) | -0.032 (0.10) |
| PMv-SMATT | 0.057 (0.09) | 0.024 (0.06) | -0.025 (0.09) | 0.039 (0.07) | -0.046 (0.08) | 0.017 (0.05) | -0.017 (0.09) |
| SMA-SMATT | 0.094 (0.07) | 0.034 (0.08) | -0.020 (0.10) | 0.056 (0.06) | -0.044 (0.09) | 0.025 (0.08) | -0.010 (0.10) |
| preSMA-SMATT | 0.061 (0.05) | 0.025 (0.06) | -0.031 (0.09) | 0.056 (0.05) | -0.051 (0.09) | 0.012 (0.07) | -0.024 (0.09) |
| S1-SMATT | 0.033 (0.08) | 0.006 (0.06) | -0.056 (0.10) | 0.016 (0.08) | -0.069 (0.10) | 0.002 (0.06) | -0.050 (0.10) |
| Cerebello-thalamo-cortical | 0.071 (0.07) | 0.015 (0.07) | -0.033 (0.09) | 0.026 (0.09) | -0.052 (0.09) | 0.011 (0.06) | -0.025 (0.09) |
| STN to GP | 0.045 (0.07) | -0.005 (0.07) | -0.061 (0.08) | 0.033 (0.07) | 0.003 (0.06) | -0.021 (0.07) | -0.085 (0.07) |
| Nigrostriatal | 0.058 (0.09) | 0.007 (0.08) | -0.074 (0.10) | 0.047 (0.10) | -0.035 (0.11) | -0.010 (0.06) | -0.089 (0.09) |

Supplemental Table 3: FDC mean (SD) of white matter tracts showing significant group effects.

|  | Con  (n = 16) | Pre  (n = 34) | Ataxic  (n = 50) | SCA1 Pre  (n = 10) | SCA1 Ataxic  (n = 14) | SCA3 Pre  (n = 24) | SCA3 Ataxic  (n = 36) |
| --- | --- | --- | --- | --- | --- | --- | --- |
| Cerebellar ICP | 0.619 (0.06) | 0.493 (0.07) | 0.393 (0.07) | 0.511 (0.06) | 0.399 (0.08) | 0.485 (0.07) | 0.391 (0.07) |
| Cerebellar MCP | 0.652 (0.07) | 0.586 (0.08) | 0.459 (0.09) | 0.583 (0.07) | 0.448 (0.11) | 0.587 (0.08) | 0.463 (0.08) |
| Cerebellar SCP | 0.756 (0.09) | 0.689 (0.09) | 0.564 (0.11) | 0.709 (0.09) | 0.525 (0.10) | 0.681 (0.08) | 0.579 (0.11) |
| M1-SMATT | 0.612 (0.07) | 0.576 (0.06) | 0.518 (0.07) | 0.572 (0.06) | 0.488 (0.06) | 0.578 (0.06) | 0.530 (0.07) |
| PMd-SMATT | 0.578 (0.06) | 0.536 (0.05) | 0.480 (0.07) | 0.534 (0.05) | 0.456 (0.07) | 0.536 (0.06) | 0.489 (0.06) |
| PMv-SMATT | 0.541 (0.07) | 0.511 (0.05) | 0.459 (0.06) | 0.508 (0.05) | 0.431 (0.05) | 0.513 (0.05) | 0.470 (0.06) |
| SMA-SMATT | 0.636 (0.07) | 0.588 (0.06) | 0.518 (0.07) | 0.583 (0.05) | 0.491 (0.07) | 0.590 (0.06) | 0.529 (0.07) |
| preSMA-SMATT | 0.568 (0.05) | 0.545 (0.04) | 0.489 (0.05) | 0.556 (0.04) | 0.469 (0.05) | 0.540 (0.05) | 0.497 (0.05) |
| S1-SMATT | 0.575 (0.06) | 0.543 (0.06) | 0.486 (0.07) | 0.546 (0.06) | 0.463 (0.06) | 0.541 (0.06) | 0.495 (0.07) |
| Cerebello-thalamo-cortical | 0.628 (0.07) | 0.574 (0.07) | 0.523 (0.07) | 0.576 (0.07) | 0.489 (0.07) | 0.573 (0.07) | 0.536 (0.07) |
| STN to GP | 0.601 (0.09) | 0.542 (0.08) | 0.463 (0.08) | 0.550 (0.08) | 0.489 (0.07) | 0.539 (0.07) | 0.453 (0.08) |
| Nigrostriatal | 0.724 (0.12) | 0.685 (0.09) | 0.593 (0.10) | 0.708 (0.11) | 0.600 (0.12) | 0.675 (0.07) | 0.591 (0.10) |
| Corticostriatal | 0.548 (0.06) | 0.522 (0.05) | 0.490 (0.06) | 0.521 (0.05) | 0.458 (0.06) | 0.522 (0.05) | 0.503 (0.06) |
| SMA-TCATT | 0.630 (0.06) | 0.597 (0.06) | 0.559 (0.07) | 0.603 (0.05) | 0.522 (0.07) | 0.594 (0.06) | 0.574 (0.07) |
| Inferior Temporal Gyrus-TCATT | 0.634 (0.07) | 0.580 (0.05) | 0.588 (0.07) | 0.580 (0.04) | 0.562 (0.04) | 0.580 (0.06) | 0.599 (0.08) |
| Middle Temporal Gyrus-TCATT | 0.659 (0.07) | 0.604 (0.05) | 0.612 (0.07) | 0.603 (0.04) | 0.585 (0.05) | 0.604 (0.05) | 0.623 (0.08) |

Supplemental Table 4: Pearson correlations between CAG repeat length, SARA, and FARS ADL and tracts showing significant FD measures.

|  | SCA1 | | | | | | SCA3 | | | | | |
| --- | --- | --- | --- | --- | --- | --- | --- | --- | --- | --- | --- | --- |
|  | CAG repeat length, long allele  (n = 23) | | SARA  (n = 24) | | FARS ADL  (n = 24) | | CAG repeat length, long allele  (n = 59) | | SARA  (n = 60) | | FARS ADL  (n = 60) | |
|  | P*_FDR_* | r | P*_FDR_* | r | P*_FDR_* | r | P*_FDR_* | r | P*_FDR_* | r | P*_FDR_* | r |
| Cerebellar ICP | 0.141 | -0.37 | **0.004** | **-0.61** | **0.0004** | **-0.67** | 0.055 | -0.34 | **7E-06** | **-0.58** | **0.0002** | **-0.52** |
| Cerebellar MCP | 0.226 | -0.28 | **0.009** | **-0.54** | 0.082 | -0.37 | 0.966 | 0.02 | **0.0001** | **-0.50** | **0.007** | **-0.40** |
| Cerebellar SCP | **0.0004** | **-0.75** | **0.0003** | **-0.75** | **0.0002** | **-0.73** | **0.001** | **-0.49** | **0.0003** | **-0.46** | **0.007** | **-0.39** |
| M1 - SMATT | 0.168 | -0.32 | **0.007** | **-0.56** | **0.0001** | **-0.76** | 0.644 | 0.11 | **0.0002** | **-0.47** | **0.010** | **-0.36** |
| PMd - SMATT | 0.144 | -0.36 | **0.021** | **-0.48** | **0.0001** | **-0.74** | 0.966 | 0.02 | **0.0002** | **-0.47** | **0.010** | **-0.36** |
| PMv - SMATT | 0.114 | -0.43 | **0.004** | **-0.61** | **0.0004** | **-0.68** | 0.286 | 0.21 | **6E-05** | **-0.51** | **0.031** | **-0.30** |
| SMA - SMATT | 0.141 | -0.38 | **0.021** | **-0.48** | **0.0004** | **-0.68** | 0.644 | 0.12 | **2.4E-07** | **-0.65** | **0.007** | **-0.38** |
| preSMA - SMATT | 0.147 | -0.35 | **0.0004** | **-0.73** | **0.0004** | **-0.67** | 0.289 | 0.19 | **1.8E-06** | **-0.61** | **0.002** | **-0.46** |
| S1 - SMATT | 0.141 | -0.39 | **0.004** | **-0.60** | **9.7E-05** | **-0.78** | 0.802 | 0.06 | **0.005** | **-0.37** | **0.021** | **-0.32** |
| Cerebello-thalamo-cortical | **0.002** | **-0.69** | **0.008** | **-0.55** | **0.0001** | **-0.74** | 0.988 | 0.00 | 0.078 | -0.23 | 0.081 | -0.24 |
| STN to GP | 0.462 | -0.17 | 0.294 | -0.22 | 0.082 | -0.37 | 0.802 | 0.07 | **2.1E-05** | **-0.54** | **0.036** | **-0.29** |
| PMd - TCATT | 0.114 | -0.44 | **0.004** | **-0.60** | **0.0004** | **-0.67** | 0.289 | 0.19 | **0.0006** | **-0.44** | 0.289 | -0.15 |
| SMA - TCATT | 0.114 | -0.43 | **0.001** | **-0.68** | **0.0004** | **-0.68** | 0.183 | 0.27 | **2.1E-05** | **-0.54** | 0.053 | -0.26 |
| Supramarginal Gyrus - TCATT | 0.655 | -0.10 | **0.046** | **-0.42** | 0.106 | -0.34 | 0.232 | 0.24 | 0.128 | -0.20 | 0.808 | -0.03 |

BOLD text indicates P_FDR_ < 0.05.

Supplemental Table 5: Pearson correlations between CAG repeat length, SARA, and FARS ADL and tracts showing significant FC measures.

|  | SCA1 | | | | | | SCA3 | | | | | |
| --- | --- | --- | --- | --- | --- | --- | --- | --- | --- | --- | --- | --- |
|  | CAG repeat length, long allele  (n = 23) | | SARA  (n = 24) | | FARS ADL  (n = 24) | | CAG repeat length, long allele  (n = 59) | | SARA  (n = 60) | | FARS ADL  (n = 60) | |
|  | P*_FDR_* | r | P*_FDR_* | r | P*_FDR_* | r | P*_FDR_* | r | P*_FDR_* | r | P*_FDR_* | r |
| Cerebeller ICP | **0.041** | **-0.52** | **0.0003** | **-0.73** | **6.9E-05** | **-0.77** | 0.191 | -0.26 | **6.6E-06** | **-0.59** | **0.0003** | **-0.51** |
| Cerebellar MCP | 0.209 | -0.32 | **0.004** | **-0.63** | **0.0006** | **-0.68** | 0.312 | -0.19 | **6.6E-06** | **-0.58** | **0.0003** | **-0.49** |
| Cerebellar SCP | **0.006** | **-0.67** | **0.0002** | **-0.76** | **8.6E-06** | **-0.82** | **0.0004** | **-0.51** | **5.7E-05** | **-0.53** | **0.0003** | **-0.50** |
| M1 - SMATT | 0.168 | -0.35 | 0.081 | -0.37 | **0.003** | **-0.60** | 0.863 | -0.04 | 0.060 | -0.27 | **0.022** | **-0.31** |
| PMd - SMATT | 0.168 | -0.38 | **0.033** | **-0.47** | **0.0003** | **-0.71** | 0.863 | -0.06 | 0.120 | -0.21 | **0.022** | **-0.31** |
| PMv - SMATT | 0.363 | -0.21 | 0.073 | -0.40 | **0.003** | **-0.60** | 0.894 | -0.02 | 0.207 | -0.17 | 0.076 | -0.23 |
| SMA - SMATT | 0.168 | -0.36 | **0.028** | **-0.50** | **0.0002** | **-0.72** | 0.863 | -0.04 | 0.152 | -0.19 | **0.028** | **-0.29** |
| preSMA - SMATT | 0.295 | -0.25 | **0.026** | **-0.51** | **0.0004** | **-0.69** | 0.749 | -0.10 | 0.110 | -0.23 | **0.019** | **-0.33** |
| S1 - SMATT | 0.168 | -0.36 | 0.078 | -0.38 | **0.004** | **-0.57** | 0.863 | -0.04 | **0.030** | **-0.31** | **0.019** | **-0.33** |
| Cerebello-thalamo-cortical | **0.034** | **-0.56** | 0.066 | -0.41 | **0.0008** | **-0.66** | 0.312 | -0.19 | 0.107 | -0.23 | **0.019** | **-0.33** |
| STN to GP | 0.737 | -0.07 | 0.135 | -0.31 | 0.187 | -0.28 | 0.147 | -0.29 | **0.0001** | **-0.50** | **0.006** | **-0.39** |
| Nigrostriatal | 0.295 | -0.26 | **0.026** | **-0.51** | **0.020** | **-0.48** | 0.312 | -0.19 | **0.0001** | **-0.50** | **0.022** | **-0.30** |

BOLD text indicates P_FDR_ < 0.05.

Supplemental Table 6: Pearson correlations between CAG repeat length, SARA, and FARS ADL and tracts showing significant FDC measures.

|  | SCA1 | | | | | | SCA3 | | | | | |
| --- | --- | --- | --- | --- | --- | --- | --- | --- | --- | --- | --- | --- |
|  | CAG repeat length, long allele  (n = 23) | | SARA  (n = 24) | | FARS ADL  (n = 24) | | CAG repeat length, long allele  (n = 59) | | SARA  (n = 60) | | FARS ADL  (n = 60) | |
|  | P*_FDR_* | r | P*_FDR_* | r | P*_FDR_* | r | P*_FDR_* | r | P*_FDR_* | r | P*_FDR_* | r |
| Cerebellar ICP | 0.075 | -0.50 | **0.0002** | **-0.75** | **4.9E-05** | **-0.76** | 0.131 | -0.31 | **4.7E-07** | **-0.63** | **3.68E-05** | **-0.55** |
| Cerebellar MCP | 0.117 | -0.37 | **0.0004** | **-0.72** | **0.0005** | **-0.67** | 0.752 | -0.15 | **3.7E-08** | **-0.68** | **3.68E-05** | **-0.56** |
| Cerebellar SCP | **0.0005** | **-0.76** | **2.1E-05** | **-0.81** | **2.4E-05** | **-0.81** | **0.0001** | **-0.55** | **3.1E-05** | **-0.54** | **0.0003** | **-0.49** |
| M1 - SMATT | 0.117 | -0.39 | **0.015** | **-0.52** | **0.0002** | **-0.70** | 0.984 | 0.00 | **0.0009** | **-0.44** | **0.004** | **-0.40** |
| PMd - SMATT | 0.117 | -0.40 | **0.012** | **-0.54** | **4.9E-05** | **-0.77** | 0.984 | -0.04 | **0.004** | **-0.38** | **0.004** | **-0.39** |
| PMv - SMATT | 0.131 | -0.35 | **0.008** | **-0.59** | **0.0002** | **-0.71** | 0.984 | 0.06 | **0.002** | **-0.42** | **0.007** | **-0.36** |
| SMA - SMATT | 0.117 | -0.41 | **0.010** | **-0.57** | **4.9E-05** | **-0.76** | 0.984 | 0.01 | **0.0007** | **-0.45** | **0.004** | **-0.39** |
| preSMA - SMATT | 0.131 | -0.35 | **0.0005** | **-0.70** | **4.9E-05** | **-0.78** | 0.984 | 0.02 | **0.0002** | **-0.49** | **0.0003** | **-0.49** |
| S1 - SMATT | 0.117 | -0.41 | **0.012** | **-0.55** | **0.0002** | **-0.70** | 0.984 | -0.02 | **0.002** | **-0.41** | **0.004** | **-0.39** |
| Cerebello-thalamo-cortical | **0.014** | **-0.62** | **0.012** | **-0.54** | **0.0002** | **-0.72** | 0.752 | -0.15 | **0.008** | **-0.35** | **0.007** | **-0.36** |
| STN to GP | 0.311 | -0.24 | 0.084 | -0.37 | **0.025** | **-0.46** | 0.984 | -0.08 | **1.3E-06** | **-0.61** | **0.004** | **-0.39** |
| Nigrostriatal | 0.117 | -0.38 | **0.019** | **-0.50** | **0.008** | **-0.54** | 0.984 | 0.02 | **8.6E-06** | **-0.57** | **0.010** | **-0.34** |
| Corticostriatal | 0.117 | -0.39 | 0.084 | -0.37 | **0.0007** | **-0.65** | 0.984 | -0.01 | 0.178 | -0.19 | 0.076 | -0.24 |
| SMA - TCATT | 0.117 | -0.44 | **0.030** | **-0.46** | **4.9E-05** | **-0.77** | 0.932 | 0.11 | 0.287 | -0.15 | 0.113 | -0.22 |
| Inferior Temporal Gyrus - TCATT | 0.982 | 0.00 | 0.570 | -0.12 | 0.165 | -0.29 | 0.752 | 0.16 | 0.393 | 0.11 | 0.987 | 0.00 |
| Middle Temporal Gyrus - TCATT | 0.958 | -0.03 | 0.570 | -0.12 | 0.099 | -0.35 | 0.752 | 0.14 | 0.390 | 0.12 | 0.848 | -0.03 |

BOLD text indicates P_FDR_ < 0.05.
